# Membrane Localization of Membrane Type 1 Matrix Metalloproteinase by CD44 Regulates the Activation of Pro-Matrix Metalloproteinase 9 in Osteoclasts

**DOI:** 10.1155/2013/302392

**Published:** 2013-07-28

**Authors:** Meenakshi A. Chellaiah, Tao Ma

**Affiliations:** Department of Oncology and Diagnostic Sciences, Dental School, University of Maryland, Baltimore, MD 21201, USA

## Abstract

CD44, MT1-MMP, and MMP9 are implicated in the migration of osteoclast and bone resorption. This study was designed to determine the functional relationship between CD44 and MT1-MMP in the activation of pro-MMP9. We used osteoclasts isolated from wild-type and CD44-null mice. Results showed that MT1-MMP is present in multiple forms with a molecular mass ~63, 55, and 45 kDa in the membrane of wild-type osteoclasts. CD44-null osteoclasts demonstrated a 55 kDa active MT1-MMP form in the membrane and conditioned medium. It failed to activate pro-MMP9 because TIMP2 binds and inhibits this MT1-MMP (~55 kDa) in CD44-null osteoclasts. The role of MT1-MMP in the activation of pro-MMP9, CD44 expression, and migration was confirmed by knockdown of MT1-MMP in wild-type osteoclasts. Although knockdown of MMP9 suppressed osteoclast migration, it had no effects on MT1-MMP activity or CD44 expression. These results suggest that CD44 and MT1-MMP are directly or indirectly involved in the regulation of pro-MMP9 activation. Surface expression of CD44, membrane localization of MT1-MMP, and activation of pro-MMP9 are the necessary sequence of events in osteoclast migration.

## 1. Introduction

Matrix metalloproteinases (MMPs) are a group of endopeptidases that regulate osteoclast migration and bone resorption [1–3]. Proteinases mobilize bone matrix proteins and determine where and when bone resorption should be initiated. MMP9 is predominantly expressed by osteoclasts and osteoclast precursors in adult bone [4]. MMP9 has been proven to be indispensable for the migration of osteoclasts through collagen both in periosteum and developing marrow cavity of primitive long bones [5]. Long bones of MMP9-knockout mice are 10% shorter than bones from wild-type mice [6]. Osteoblasts express MMP2, and bones of MMP2-knockout mice are lower in bone mineral density than those of wild-type mice. Observations in these knockout mice suggest that MMP2 and MMP9 may have a compositional and structural influence, respectively, on the biomechanical properties of whole bones.

MT1-MMP was found to be highly expressed in purified osteoclasts as compared with alveolar macrophages, bone stromal cells, and various other cell types [7]. The localization of MT1-MMP was shown in the sealing zone of osteoclast in vivo. Its distribution suggests that this enzyme modifies the bone surface to facilitate the migration and attachment of osteoclasts as well as to scavenge the resorption lacunae [8]. CD44 is a cell surface molecule, originally identified as a receptor for hyaluronic acid [9] and later found to have affinity to several matrix components including osteopontin, collagen, fibronectin, and matrix metalloproteinases (MMPs) [10–15]. Cell surface colocalization of MT1-MMP and CD44 at the leading edge of migratory cells at lamellipodia suggests the possibility that the MT1-MMP may be involved in the processing of CD44 receptor [16].

We have previously shown that CD44 standard (CD44s) is the most abundantly expressed isoform in osteoclasts [17, 18]. Cao et al. have demonstrated that the tibia of CD44^−/−^ mice was shorter. The cortical bone was thicker and medullary area was smaller [19]. In vitro studies exhibited a significant decrease in bone resorption by osteoclasts from CD44^−/−^ mice. A decrease in bone resorption activity of CD44^−/−^ osteoclasts is due to reduced motility [20] which resulted in a mild osteopetrotic phenotype [19]. Surface expression of CD44 can influence signaling pathways that are critical for the activation of MMPs and motility [3, 21, 22].

The proteolytic activities of MMPs are regulated by their endogenous inhibitors, the tissue inhibitor of metalloproteinases (TIMPs) [23, 24]. TIMP2, -3, and -4 are shown as strong inhibitors of MT1-MMP [25, 26]. Increased staining for TIMP2 was also observed in association with increased synthesis of MMP2 and MMP9 [27]. MT1-MMP mediated activation of MMP2 has been shown to require the assistance of TIMP2 on the cell surface [28]. The components of the trimolecular complex MT1-MMP/TIMP2/pro-MMP2 regulate MMP2 activation. It has been shown that increased activation of MT1-MMP/MMP2 complex also activates pro-MMP9 [29]. Although MMP2 is expressed by osteoclasts, the secretory level and activity of MMP2 are significantly lower in the conditioned media of osteoclasts than WT osteoclasts [20]. Although MMP9 has been shown to be involved in the migration of osteoclasts, the mechanism of its activation remains unknown. Recent studies have shown the activation of pro-MMP9 by an MT1-MMP associated protein through RhoA and actin remodeling [30]. 

 Earlier studies from our laboratory showed that CD44 surface expression is regulated by actin remodeling through RhoA activation. CD44 deficiency in osteoclasts increases the secretion of MT1-MMP and reduces activation of pro-MMP9 [20]. We hypothesize that activation of pro-MMP9 requires surface localization of MT1-MMP/CD44s complex. Our studies with CD44-null osteoclasts indicate that CD44 functions as a docking molecule for MT1-MMP. MT1-MMP localized on the membrane may function in the activation of pro-MMP9 as well as proteolytic processing and expression of CD44.

## 2. Materials and Methods

### 2.1. Materials

MMP antibody microarray was bought from Ray Biotech Inc. (Norcross, CA., USA, Cat. number: HO149801) Antibodies to CD44, MT1-MMP (goat-polyclonal), TIMP-2, and MMP9 were purchased from Santa Cruz Laboratory. MT1-MMP activation assay kit was bought from Amersham (Cat. number: RPN 2637). MT1-MMP antibody (MAB3329) was also purchased from Chemicon. GAPDH antibody was bought from Abcam Inc (Cambridge, MA., USA). CY2- or CY3-conjugated secondary antibodies were purchased from Jackson ImmunoResearch Laboratories, Inc. (West Grove, PA, USA). MCSF-1 was bought from R&D Systems, Inc. (Minneapolis, MN, USA). All other chemicals were purchased from Sigma (St. Louis, MO, USA).

### 2.2. Preparation of Osteoclast Precursors from Mice

Wild-type (WT) and CD44-null (CD44^−/−^) mice in a C57/BL6 background were used for osteoclast preparation. CD44^−/−^ mice generated by Dr. Tak W. Mak (Ontario Cancer Institute, Toronto, ON, Canada) were used [32]. WT and CD44^−/−^ mice were generated in the animal facility of the University of Maryland Dental School. Breeding and maintenance were carried out as per the guidelines and approval of the institutional animal care and use committee. WT mice were also bought from Harlan Laboratory on occasion. Osteoclasts were generated in vitro using mouse bone marrow cells as described previously [18]. 

### 2.3. RNA Interference-Mediated Silencing of Endogenous Protein

Transfection of small interfering RNA (SiRNA) sequences for targeting endogenous MT1-MMP and MMP9 was carried out using the MIRUS transfection reagent according to the manufacturer's instructions as described previously [33]. From six sequences tested, the SiRNA sequences 5′-GAAGCCUGGCUACAGCAAUAU-3′ reduced the expression of MT1-MMP most efficiently. The 5′-GGUCCAUGCUGCAGAAAAACU-3′ scrambled RNA (ScRNAi) sequence was used as a control. Sense (5′-GGCAUACUUGACCGCUAUTT-3′) and SiRNA (5′-AUAGCGGUACAAGUAGCCTC-3′) sequences for MMP9 were made from Ambion, Inc. (Austin, TX, USA). Transfected SiRNA concentrations were 50 nM for MT1-MMP and 40 nM for MMP9. The effect of different SiRNA (Mt1-MMP or MMP9) on the cellular levels of CD44 is shown in supplementary Figure 1 (see Supplementary Material available online at http://dx.doi.org/10.1155/2013/302392). Nontargeting scrambled nucleotides were used at matching concentrations. Cell viability was tested by trypan blue exclusion test, and it was found that treatment with indicated SiRNA or ScRNAi had no significant effect on cell viability.

### 2.4. Preparation of Total Cellular and Membrane Lysates

Following various treatments, osteoclasts were washed three times with cold PBS and lysed in a radio immune precipitation (RIPA) buffer to make total cellular lysates as described previously [17]. To prepare the cell membrane lysates, osteoclasts from wild-type and CD44^−/−^ mice were washed three times with cold PBS and removed from the plates by gentle scraping with cold PBS. Cells were pelleted and resuspended in 2 mL of a buffer containing 250 mM sucrose, 20 mM Tris-HCl, and 1 mM EDTA pH 7.8. Lysis buffer was supplemented with EDTA-free complete mini-protease-inhibitor-cocktail (1 tablet per 10 mL buffer) immediately before use. Osteoclasts were homogenized in a hand homogenizer with 20–25 strokes. The homogenate was transferred to a 10 mL oak ridge centrifuge tube, and volume was made up to 10 mL close to the top of the tube. Tubes were centrifuged at 41,500 rcf (20,000 rpm) for 10 min at 4°C to pellet membranes. Pellet was washed three times with a buffer containing 100 mM sucrose, 10 mM Tris-HCl pH 7.5, 5 mM MgCl_2_, and 50 mM NaCl. Subsequently, pellet was lysed in a cold RIPA buffer.

### 2.5. Preparation of Conditioned Media from WT and CD44^−/−^ Osteoclasts

Mature WT and CD44^−/−^ osteoclast cultures were kept in serum-free medium for 24–36 h. The conditioned media were collected and centrifuged to remove cell debris. Media were concentrated (~20X concentrated) using a Centricon concentrator (Amicon, Beverly, MA, usa) and quantitated as described previously [34].

### 2.6. Fluorescence-Activated Cell Sorting (FACs) Analysis and Cell Surface Labeling by Biotinylation

FACs analysis was performed essentially as described previously [17]. Cells were labeled with NHS-biotin according to the manufacturer's guidelines (Pierce, Rockford, IL, USA). Briefly, cells were incubated with 0.5 mg/mL biotin for 30–40 min at 4°C and washed three times with cold PBS. Cells were lysed with RIPA lysis buffer. Equal amounts of protein lysates were used for immunoprecipitation with an antibody to MT1-MMP. The immune complexes were adsorbed onto streptavidin agarose which precipitates the biotinylated MT1-MMP protein from the cell surface as described previously [17]. These complexes were used to determine the activity of membrane associated MT1-MMP enzyme.

### 2.7. MT1-MMP and MMP9 Activity Assays

Conditioned medium or immune complexes pulled down with streptavidin agarose were used for MMP-9 (BIOMOL; Cat. Number: AK410) and MT1-MMP activity assay (Amersham; Cat. number: RPN 2637) according to the manufacturer's recommendations and as previously described [34].

### 2.8. Gelatin Zymography and Reverse Zymography of Conditioned Media from Osteoclasts

Gelatin zymography of culture media (~25–30 *μ*g protein) or membrane fraction (~40–50 *μ*g protein) was performed as described previously [20, 35]. The reverse zymogram was done to digest the gelatin (2.25%) present in the gels as described previously [36] with minor modifications. Zymogram gels containing 2.25% gelatin were incubated in the conditioned medium collected from WT osteoclasts (20X concentrated; ~50 *μ*g protein) or 150 ng/mL progelatinase A or B for 12–15 h at 37°C. Conditioned medium provides a source of activated gelatinases which are able to degrade gelatin in the gel extensively and more efficiently within 8–10 h at 37°C. After proteolysis, gels were then stained with a staining solution (0.025% Coomassie brilliant blue in 50% methanol and 7% acetic acid) for 1-2 h at RT. Gels were then destained by washing three times in a destaining solution (40% methanol, 10% acetic acid, and 50% distilled water) for 1-2 h at RT. TIMP2 was observed as dark bands against a lighter background. 

### 2.9. Transwell Migration Assay

Transwell cell migration assay was performed in transwell migration chambers (8-*μ*m pore size; Costar) with osteoclasts from wild-type mice as described previously [37]. Osteoclasts (2 × 10^4^ cells) were added to the upper chamber containing the membrane and allowed to adhere for 2–3 h at 37°C. After the cells attached to the membrane, the culture medium (100 mL) was replaced with the following reagents at the indicated concentrations in the presence of RANKL and mCSF-1: Si and scrambled RNAi for MT1-MMP (50 nM) and MMP9 (40 nM; [38]), GM6001 (15 *μ*M; [20]), and TIMP1 or TIMP2 (100 ng/mL; [39]). Counting of the migrated osteoclasts to the underside of the membrane was performed as described previously [37].

### 2.10. Immunoprecipitation and Immunoblotting Analysis

Equal amount of membrane proteins (50 or 100 *μ*g) or conditioned media (25 *μ*g) was used for immunoprecipitation with antibodies of interest. Immune complexes from the immunoprecipitates as well as total cellular lysate and conditioned media proteins were subjected to SDS-PAGE and immunoblotting analyses as described previously {Chellaiah, 1996 366/id}.

### 2.11. Immunostaining and Gelatin Matrix Degradation Assay

Matrix degradation assay was done using cross-linked fluorescein isothiocyanate (FITC)-conjugated gelatin matrix-coated cover slips as described previously {Desai, 2008 7105/id}. Osteoclasts cultured on gelatin matrix or glass cover slips were immunostained with MT1-MMP and MMP9 as described previously [40]. Immunostained osteoclasts on cover slips or gelatin matrix (green) (red) were scanned and imaged with a Bio-Rad confocal laser scanning microscope. Images were stored in TIF format and processed by using Photoshop (Adobe Systems, Inc., Mountain View, CA, USA).

### 2.12. Data Analysis

All comparisons were made as “% control,” which refers to vehicle or scrambled RNAi treated and untreated cells. The other treatment groups in each experiment were normalized to each control value. A value of <0.05 was considered significant. Data presented are means ± SEM of experiments done at different times normalized to intraexperimental control values. For statistical comparisons, analysis of variance (ANOVA) was used with the Bonferonni corrections (Instat for IBM, version 2.0; GraphPad Software, San Diego, CA, USA).

## 3. Results

### 3.1. CD44 Knockdown Reduces MT1-MMP Localization in the Membrane of Osteoclasts

**Figure 1:**
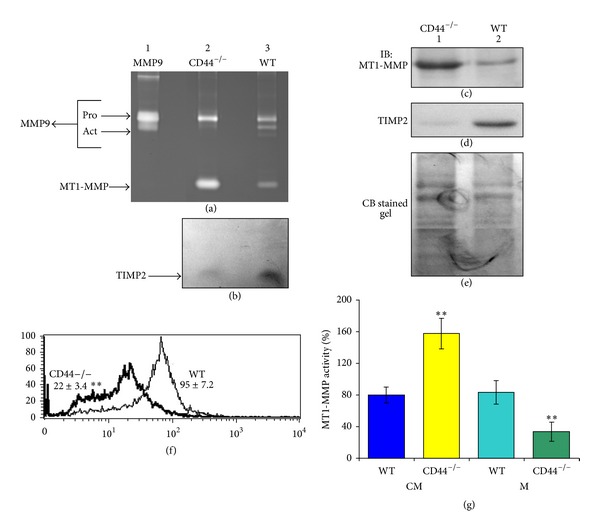
CD44 knockout reduces the levels of active MMP9 but increases MT1-MMP in osteoclasts. (a) *Zymogram analysis*. Conditioned media from CD44^−/−^ (lane 2) and WT osteoclasts (lane 3) were analyzed by zymogram analysis. Recombinant MMP9 protein (UBI) was used as an identification marker. (b) *Reverse zymography*. The lower half of the zymogram was cut and incubated with culture media to digest the gelatin in the gel. Subsequently, the gel was stained with Coomassie blue to detect TIMP2 protein as described in the Methods-section. Dark TIMP2 band (indicated by arrows) was observed in the light background. (c–e) *Immunoblotting analyses*. Soluble or secreted levels of MT1-MMP and TIMP 2 in the conditioned media of CD44^−/−^ (lane 1) and WT (lane 2) osteoclasts were determined by immunoblotting analysis with relevant antibody in succession after stripping the blot. Finally the blot was stained with a Coomassie blue stain to determine the amount of protein loaded in each lane (e). (f) and (g) *FACs analysis (f) and MT1-MMP activity assay (g)*. (f) A representative histogram is shown for CD44^−/−^ and WT osteoclasts. Each FACs analysis was performed in quadruplicates. Mean fluorescence intensity of MT1-MMP from two different osteoclast preparations is shown in Figure 1(f). (g) Concentrated conditioned media and MT1-MMP immunoprecipitates pulled down with streptavidin agarose were subjected to the MT1-MMP activity assay to determine the soluble and cell surface (membrane) associated enzyme activity, respectively. **P < 0.01 versus WT osteoclasts in (f) and (g). Results shown in (g) are representative of three different experiments with three different osteoclast preparations.

As shown previously, gelatin zymogram analysis demonstrates an increase in the activity of MT1-MMP in the conditioned medium of CD44^−/−^ osteoclasts (Figure 1(a), lane 2) as compared with WT osteoclasts (lane 3) [20]. Activation of pro-MMP9 to active MMP9 is decreased considerably in the conditioned medium of CD44^−/−^ osteoclasts (Figure 1, lane 2). WT osteoclasts exhibited both pro- (92 kDa) and active (84 kDa) MMP9 (Figure 1, lane 3). WT or CD44^−/−^ osteoclasts failed to show detectable levels of MMP2 activity in the zymogram analysis. Reverse zymogram analysis of the lower half of the zymogram gel demonstrated a protein with molecular mass ~24–27 kDa (Figure 1(b)) which was confirmed as TIMP2 in immunoblotting analysis (Figure 1(d)). Secretion of TIMP2 is more in WT osteoclasts (lane 3 in (b) and lane 2 in (d)). Immunoblotting analysis of the conditioned medium (c) with an MT1-MMP antibody confirmed that the increased activity of MT1-MMP in the conditioned media is due to an increase in the secreted or soluble MT1-MMP levels in CD44^−/−^ osteoclasts (Figure 1(c), lane 1).

FACs analysis demonstrated a significant decrease in the surface levels of MT1-MMP in CD44^−/−^ osteoclasts. This suggests that CD44 may have a role in the membrane localization of MT1-MMP (f). An increase in MT1-MMP activity in the conditioned medium of CD44^−/−^ osteoclasts in an in vitro assay (Figure 1(g)) supports the zymogram analysis shown in (a). Furthermore, MT1-MMP activity concurs with the MT1-MMP levels in the conditioned medium (a and c) and membrane (f) in WT and CD44^−/−^ osteoclasts.

### 3.2. Membrane Localization of MT1-MMP (~63–65 kDa) Protein Is Significantly Reduced in CD44^−/−^ Osteoclasts

**Figure 2:**
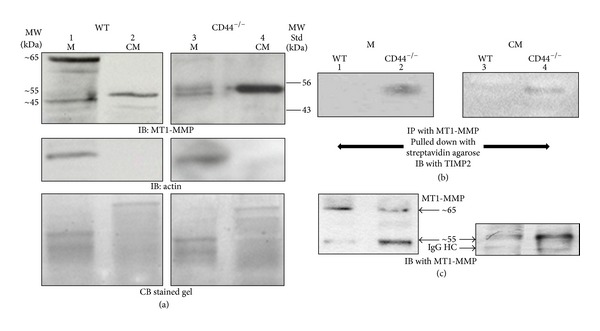
CD44 knockdown reduces membrane localization of MT1-MMP (~63 kDa) in osteoclasts. (a) Membrane fraction (M; lanes 1 and 3) and conditioned medium (CM; lanes 2 and 4) of osteoclasts isolated from WT (lanes 1 and 2) and CD44^−/−^ (lanes 3 and 4) mice were analyzed by immunoblotting with an MT1-MMP antibody (top panel). Well-characterized protein standards (indicated on the right side of the figure) were used to estimate the approximate molecular weight of MT1-MMP in the membrane fraction and conditioned media. Estimated approximate molecular masses of MT1-MMP (~65, 55, and 45) are indicated on the left side of the figure. Membranes were stripped successively and blotted with an antibody to actin (middle panels) and stained with Coomassie blue (bottom panels) as loading controls for membrane fraction and conditioned medium, respectively. Data shown are representative of three independent experiments with similar results. (b) and (c) TIMP2 binds active MT1-MMP form (~55 kDa) in the membrane and conditioned medium of CD44^−/−^ osteoclasts. Equal amount of protein from membrane fraction (50 *μ*g; M) and conditioned medium (25 *μ*g; CM) from WT (lanes 1 and 3) and CD44^−/−^ (lanes 2 and 4) was used for immunoprecipitation with an antibody to MT1-MMP and immunoblotted (IB) with an antibody to TIMP2 (top panels in (b)). Membranes were stripped and blotted with an antibody to MT1-MMP ((c); bottom panels). Protein A-HRP was used as secondary antibody as described previously [31]. This analysis provided specific signals corresponding to 55 kDa. Data shown are representative of three independent experiments with similar results.

MT1-MMP exists in different forms. Therefore, we investigated whether CD44 would serve to maintain the membrane localization of any of the forms of the MT1-MMP. Membrane lysate fraction (Figure 2, lanes 1 and 3) and conditioned medium (lanes 2 and 4) of WT and CD44^−/−^ osteoclasts were subjected to immunoblotting analysis with an MT1-MMP antibody. Membrane fraction of WT osteoclasts demonstrated latent (~63–65 kDa) and catalytically inactive (~45 kDa) forms of MT1-MMP. Conditioned medium of WT osteoclasts demonstrated a 55 kDa protein band corresponding to the active MT1-MMP form (lane 2) and this band is ~fivefold higher in CD44^−/−^ osteoclasts (lane 4). These osteoclasts also demonstrated the presence of ~55 and ~45 kDa MT1-MMP forms in the membrane fraction (lane 3). However, the level of 55 kDa is considerably lower than the levels observed in the conditioned medium. The presence of extremely weak amount of ~63 kDa MT1-MMP form in the membrane fraction of CD44^−/−^ osteoclasts suggests a role for CD44 in the membrane localization of MT1-MMP. The mechanism of formation of ~55 kDa MT1-MMP needs further elucidation. 

### 3.3. TIMP2 Makes Complex with Membrane-Bound and Secreted Active Form of 55 kDa MT1-MMP

Previous studies showed that TIMP2 interacts with the membrane-tethered MT1-MMP with its catalytic domain and inhibits its activity [41]. To determine interaction of TIMP2 with membrane-associated MT1-MMP, we used lysates made from WT and CD44^−/−^ osteoclasts surface labeled with NHS-biotin. Lysates were immunoprecipitated with an antibody to MT1-MMP and pulled down with streptavidin agarose. Conditioned medium from these osteoclasts was also used for immunoprecipitation with an antibody to MT1-MMP. Co-precipitation of TIMP2 with MT1-MMP was observed in the membrane (Figure 2(b), lane 2) and conditioned medium (lane 4) of CD44^−/−^ osteoclasts. Coprecipitation of TIMP2 with MT1-MMP is more in the membrane fraction regardless of the levels of MT1-MMP in the membrane (Figure 2(c)). Membranes shown in Figure 2(b) were stripped and blotted with an antibody to MT1-MMP (Figure 2(c)). As shown in Figure 2(a), 65 kDa protein band corresponding to latent MT1-MMP is more in WT than CD44^−/−^ osteoclasts. Consistently, 55 kDa protein band corresponding to active MT1-MMP form ((b), lane 2) was found in CD44^−/−^ osteoclasts in both membrane ((c), lane 2) and conditioned medium ((c), lane 4). Latent form was found to a lesser extent in WT osteoclasts ((b), lanes 1 and 3). 

### 3.4. MT1-MMP and Not MMP9 Knockdown Reduces CD44 Expression—Immunoblotting Analysis

**Figure 3:**
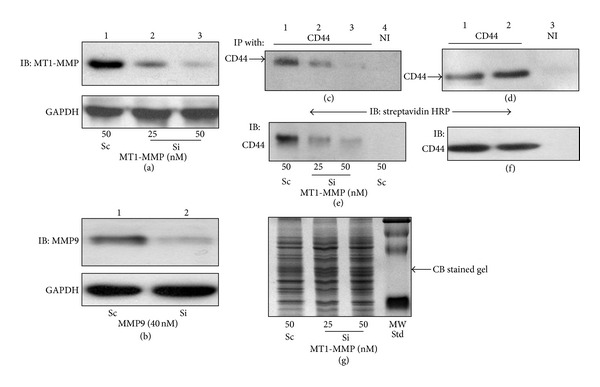
MT1-MMP knockdown reduces CD44 surface expression. (a) and (b) Immunoblotting analyses with an antibody to MT1-MMP (a) and MMP9 (b) were performed in total cellular lysates (50 *μ*g protein) made from osteoclasts treated with a scrambled RNAi (Sc; lane 1 in (a) and (b)) or SiRNA sequences to MT1-MMP (lanes 2 and 3 in (a)) and MMP9 (lane 2 in (b)). The blots in (a) and (b) was reprobed with an antibody to GAPDH after stripping (bottom panel in (a) and (b)). GAPDH level was used as a control for loading. ((c) and (d)) Immunoblotting analysis of surface expression of CD44s in osteoclasts treated with a SiRNA (Si) or scrambled RNAi (Sc) to MT1-MMP (c) and MMP9 (d). Equal amounts of proteins (50 *μ*g protein) prepared from osteoclasts treated as indicated in (c) and (d) and surface labeled with NHS-biotin were immunoprecipitated with an antibody to CD44 (lanes 1–3 in (c); lanes 1 and 2 in (d)). Lysates from scrambled RNAi-treated osteoclasts were immunoprecipitated with a nonimmune serum (NI; lane 4 in (c); lane 3 in (d)). Immunoprecipitates were probed with streptavidin-HRP to determine the surface levels of CD44 protein. Blot in (c) and (d) was stripped and reprobed with an antibody to CD44 to determine the total cellular levels of CD44 protein ((e) and (f)). (g) To ensure that equal protein amount was used for immunoprecipitation, duplicate polyacrylamide gels were run with 50 *μ*g total protein and stained with Coomassie blue staining.

Localization of MMPs to the cell membrane through their interaction with cell surface CD44 receptor has been demonstrated in different cell types including osteoclasts [22, 34, 42, 43]. The function and molecular association of MT1-MMP and CD44 at the cell surface is not known although CD44^−/−^ osteoclasts demonstrated a decrease in the membrane localization of MT1-MMP. In order to identify whether MT1-MMP or MMP9 activity is necessary for the expression of CD44, osteoclasts from WT mice were transfected with SiRNA to MT1-MMP or MMP9 (Si, Figures 3(a) and 3(b)) and incubated for 48–72 h as described previously [38]. Scrambled RNAi sequences (Sc) transfected cells were used as controls. A dose-dependent decrease in MT1-MMP protein level was observed. A maximum silencing (>80–85%) was observed at 50 nM SiRNA of MT1-MMP (Figure 3(a)) and 40 nM SiRNA of MMP9 (Figure 3(b), lane 2). 

Osteoclasts were surface labeled with NHS-biotin and equal amounts of lysate proteins (50 *μ*g) were immunoprecipitated with an antibody to sCD44 or nonimmune IgG (NI; lane 4 in (c) and lane 3 in (d)). Immunoprecipitates were blotted with streptavidin-HRP to determine the surface levels of CD44 ((c) and (d)). Subsequently, the blot was stripped and blotted with an antibody to sCD44 to determine the total cellular levels of CD44 protein ((e) and (f)). Surface and total cellular levels of sCD44 are reduced in a dose-dependent manner in osteoclasts transfected with MT1-MMP SiRNA (Figures 3(c) and 3(e), lanes 2 and 3) as compared with scrambled RNAi transfected cells ((c) and (e), lane 1). However, knockdown of MMP9 in osteoclasts had no effect on either the surface or total cellular levels of sCD44 ((d) and (e), lane 2). Concurrently, 50 *μ*g of total cellular protein was loaded on a separate gel for Coomassie blue staining (Figure 3(g)) which is used as a loading control. 

### 3.5. MT1-MMP Knockdown Reduces Cellular Levels of CD44 and Migration in Osteoclasts Immunostaining and Transwell Migration Analyses

**Figure 4:**
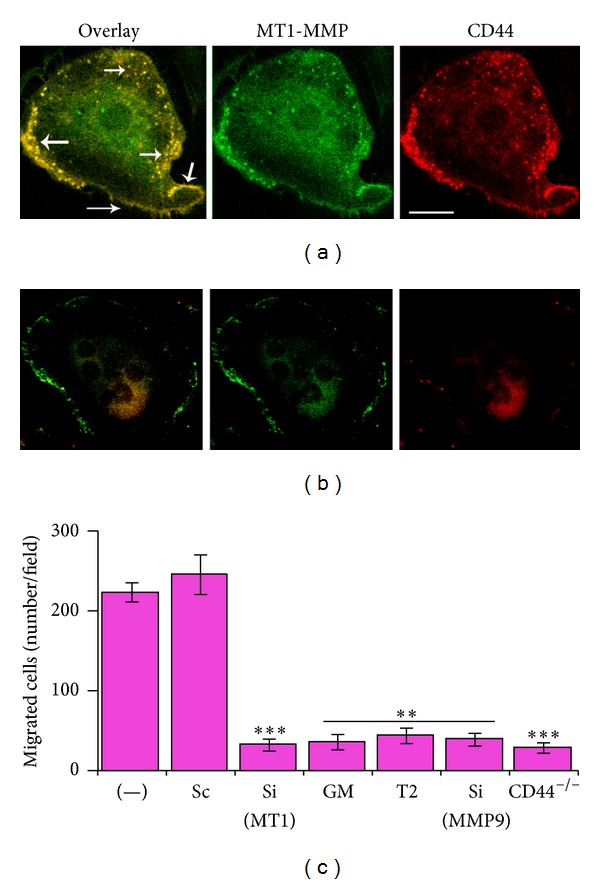
MT1-MMP and MMP 9 have roles in osteoclast migration but only MT1-MMP has a role in the expression of CD44. ((a) and (b)) Confocal microscopy analysis of osteoclasts transfected with scrambled RNAi (a) and SiRNA sequences to MT1-MMP. Osteoclasts immunostained for CD44 (red) and MT1-MMP (green) were analyzed by confocal microscopy. Colocalization (yellow) of MT1-MMP and CD44 was observed in the membrane and podosomes of osteoclasts (indicated by arrows in overlay panel). Scale bar: 50 *μ*m. (c) The effects of various treatments on the migration of WT osteoclasts were assessed using transwell migration assay. Osteoclasts from CD44^−/−^ (CD44-null) mice were also used. WT osteoclasts were treated with the following treatments: scrambled RNAi (Sc), SiRNA MT1-MMP or MMP9 (Si), MMP inhibitor GM6001 (GM), and TIMP2. Cells in ~20–25 fields were counted. Values are expressed as mean number of cells migrated/field. The experiment was repeated three times with three different osteoclast preparations. Assay was performed in quadruplicates in each experiment. Data shown are the mean ± SEM of one experiment. **P < 0.01 and ***P < 0.001 compared with (−) and Sc controls.

Consistent with the observations shown in Figure 3(e), immunostaining analysis indeed has shown a significant decrease in total cellular levels of CD44s in MT1-MMP knockdown osteoclasts (Figure 4(b)). Colocalization (yellow) of MT1-MMP (green) and CD44 (red) was observed in the podosomes (indicated by arrows in Figure 4(a)) and membrane of osteoclasts transfected with scrambled RNAi (a). A significant decrease in the expression of CD44 in MT1-MMP knockdown and CD44^−/−^ osteoclasts is associated with a significant decrease in the migration of osteoclasts in a transwell migration assay (Figure 4(c)). Migration was also affected by soluble recombinant TIMP2 and a broad spectrum MMP inhibitor GM6001. These data collectively suggest that MT1-MMP regulates the expression of CD44s. Both CD44 and MT1-MMP are involved in the migration of osteoclasts.

### 3.6. MT1-MMP Knockdown Reduces MMP9 Activity

**Figure 5:**
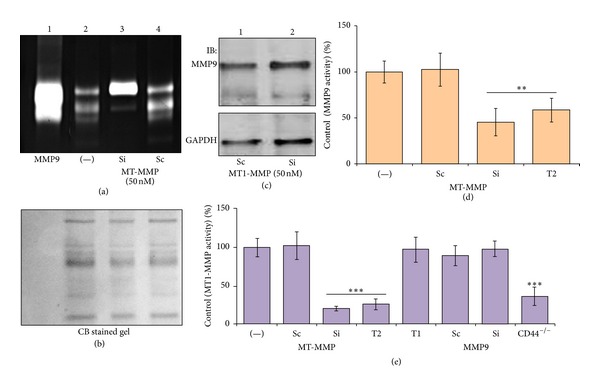
MT1-MMP regulates the activity of MMP9. (a–d) The effects of various treatments on MMP9 activity. Equal amounts of membrane proteins (50 *μ*g) from osteoclasts untransfected (−) (lane 2) or transfected with SiRNA (Si, lane 3) and ScRNAi (Sc, lane 4) to MT1-MMP were used for gelatin zymography analysis (a) and MMP9 activity assay in vitro (d). Lysates made from osteoclasts treated with TIMP2 (T2) were also used for MMP9 activity assay in vitro (d). To ensure that equal protein amount was used for zymogram analysis (a), duplicate polyacrylamide gel was run with same amount of protein and stained with Coomassie blue staining (b). Cell extracts from osteoclasts treated with ScRNAi (Sc) and SiRNA (Si) to MT1-MMP were subjected to immunoblotting analysis with an MMP9 antibody to detect total cellular levels of MMP9 ((c); top panel). Stripping and reprobing of the same blot with an antibody to GAPDH was used as loading control (bottom panel in (c)). (e) The effects of various treatments on MT1-MMP activity. MT1-MMP activity was determined in the membrane fraction of osteoclasts subjected to various treatments as indicated as follows: ScRNAi (Sc) and SiRNA (Si) to MT1-MMP and MMP9, TIMP1 (T1), and TIMP2 (T2). Untransfected and CD44-null osteoclasts are indicated as (−) and CD44^−/−^, respectively. Values in (d) and (e) are mean ± SE from three different experiments. **P < 0.05 and ***P < 0.001 compared to controls ((−) and Sc). The results shown are representative of three different experiments.

The presence of MMP9, TIMP2, and MT1-MMP in the conditioned media of osteoclasts suggests that formation of this complex may regulate the activation of pro-MMP9. Our results show that knockdown of MT1-MMP had a significant inhibitory effect on the activation of pro-MMP9 (Figure 5(a), lane 3). However, MT1-MMP knockdown had no effect on total cellular levels of MMP9 ((c), lane 2). Both pro- and active MMP9 were detected in untransfected ((a), lane 2) and ScRNAi transfected (lane 4) osteoclasts. Coomassie blue stained polyacrylamide gel was used as a loading control (b) for the zymogram gel shown in (a).

### 3.7. TIMP2 Reduces MT1-MMP and MMP9 Activities in WT Osteoclasts

TIMP2 is unique among TIMPs because it inhibits a number of MMPs besides its role in the activation. MT1-MMP initiates pro-MMP2 activation in a process that is tightly regulated by the level of TIMP2 [44]. To this end, to assess TIMP2 regulation of MT1-MMP and MMP9 activities, WT osteoclasts were treated with TIMP1 and TIMP2 (100 ng/mL for 20 h at 37°C). Osteoclasts knockdown of MT1-MMP and MMP9 with respective SiRNA was also used. Untreated (−) and scrambled RNAi treated osteoclasts were used as controls. Equal amount of membrane protein was used for MMP9 and MT1-MMP activity assay (Figure 5(d) and (e)). Basal MMP9 or MT1-MMP activity in untreated (−) osteoclasts is considered as 100%. TIMP2 reduces both MMP9 (Figure 5(d)) and MT1-MMP (Figure 5(e)) activities in osteoclasts. Under these conditions, TIMP1 did not inhibit MT1-MMP activity. MT1-MMP knockdown (Si) reduced MMP9 activity (Figure 5(d)) whereas MMP9 knockdown (Si) had no effect on MT1-MMP activity (Figure 5(e)). Consistent with the observation shown in Figure 1(g), MT1-MMP activity is significantly reduced in CD44^−/−^ osteoclasts. These data provide the evidence that MT1-MMP is a potential regulator of MMP9 activity. We show here TIMP2/MT1-MMP complex formation (Figure 2) and suppressed activity of MT1-MMP (Figure 5(e)) in the cell membrane. These findings suggest that TIMP2 is a key determinant of MT1-MMP activity. TIMP2 may inhibit MMP9 through its interaction with MT1-MMP. 

### 3.8. SiRNA to MT1-MMP Reduces Matrix Degradation

**Figure 6:**
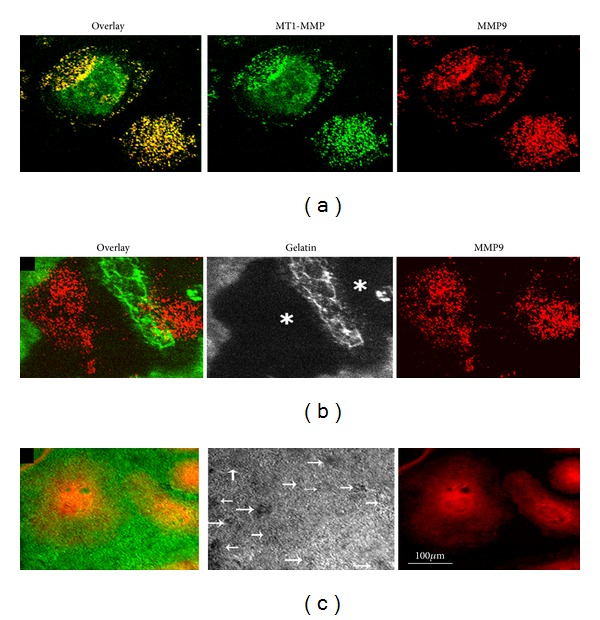
MT1-MMP knockdown reduces matrix degradation and punctate localization of MMP9 in osteoclasts. (a) Confocal microscopy analysis of distribution of MT1-MMP and MMP9 in osteoclasts cultured on glass cover slips. Immunostaining was done in nonpermeablized osteoclasts with Triton-X100. Punctate colocalization (yellow) of MT1-MMP (green) and MMP9 (red) was observed on the cell surface. (b) and (c) Confocal microscopy analysis of osteoclasts immunostained with a MMP9 antibody (red) and degradation of gelatin matrix (green). Osteoclasts transfected with scrambled RNAi (b) and SiRNA (c) sequences to MT1-MMP were subjected to gelatin degradation for 6 h. Distribution of MMP9 (red) in nonpermeablized osteoclasts and FITC-gelatin matrix (green) is shown in the overlay panels ((b) and (c)). The results represent one of three experiments performed. Scale bar: 100 *μ*m. Matrix degraded areas are indicated by asterisks in (b) and (c) (gelatin panel).

We have previously demonstrated that reducing MMP9 levels by RNA interference inhibited the degradation of matrix but not the formation of podosomes [38]. Since MT1-MMP appears to have a role in the regulation of MMP9 activity, we further determined the ability of MT1-MMP knockdown cells to degrade the gelatin matrix in vitro. First, we determined the possible interaction of MT1-MMP and MMP9 on the cell surface. Immunostaining analysis was performed in osteoclasts not permeablized with Triton X-100 as described previously [17]. Distribution is shown in a representative osteoclast (Figure 6(a)). Punctate codistribution (yellow) of MT1-MMP (green) and MMP9 (red) was observed on the surface of osteoclasts (a). 

Next, in order to determine the ability of MT1-MMP knockdown on MMP9 expression/activity and matrix degradation, osteoclasts were cultured on a matrix made of gelatin conjugated with FITC dye (green) as shown previously [38]. After incubation for 3–6 h, surface distribution of MMP9 was determined by immunostaining with an antibody to MMP9. Confocal microscopy analysis demonstrated punctate distribution of MMP9 (Figure 6(b), MMP9) on the surface as shown in (a). Matrix degradation (indicated by asterisks in gelatin panel) was observed underneath and around the osteoclasts ((b); overlay). Osteoclasts transfected with SiRNA against MT1-MMP failed to demonstrate either punctate distribution of MMP9 on the cell surface or degradation of the gelatin matrix (Figure 6(c)). Diffused distribution of MMP9 was observed on the cell surface ((c); MMP9). MT1-MMP knockdown had no effect on the total cellular levels of MMP 9 (Figure 5) but affected distribution and activity of MMP9.

### 3.9. Reducing the Levels of CD44, MT1-MMP, and MMP9 Attenuates OC Migration but Not Polarization, Sealing Ring Formation, and Bone Resorption

**Figure 7:**
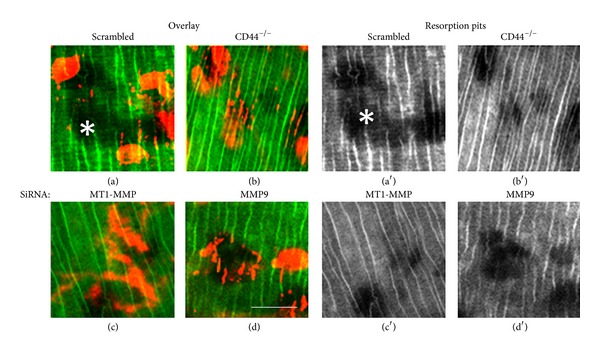
Reducing the levels of CD44, MT1-MMP, and MMP9 attenuates OC migration but not polarization, sealing ring formation, and bone resorption. Confocal microscopy analysis of distribution of actin in indicated osteoclasts cultured on dentine slices. Osteoclasts were stained for actin (red; (a–d)). Resorption pits underneath the osteoclasts are shown separately in (a′)–(d′). Dentine slices are shown in green by the reflected light. Experiments were repeated two times. Three dentine slices were used for each condition in both experiments. Results represent one of the two experiments and dentine slices performed with two different osteoclast preparations. Scale bar: 25 *μ*m.

We have previously demonstrated localization of MMP9 and actin in sealing rings of resorbing osteoclasts [20]. We next proceeded to determine if SiRNA to MT1-MMP or MMP9 has the potential to attenuate sealing ring formation and bone resorption. After treating with Sc RNAi and indicated SiRNA for 17–20 h (Figure 7), osteoclasts were cultured on dentine slices for 8–12 h. Cells were stained for actin with rhodamine phalloidin. Diffuse staining of actin is due to intense actin staining in sealing ring and cell membrane. Wild-type osteoclasts treated with a ScRNAi demonstrated convoluted multiple overlapping pits (Figures 7(a) and 7(a′)). Simple resorption pits were observed in CD44^−/−^ osteoclasts and wild-type osteoclasts treated with an SiRNA against MT1-MMP or MMP9 (Figures 7(b)–7(d); 7(b′)–7(d′)). Osteoclast migration, adhesion, polarization, and resorption are very much coupled processes. Formation of simple pits in these osteoclasts (b–d; b′–d′) suggests that these osteoclasts are not defective in adhesion, polarization, and resorption but defective in migration. 

## 4. Discussion

It has been suggested that MT1-MMP modifies the bone surface to facilitate the migration and attachment of osteoclasts [8]. Immunostaining analysis in osteoclasts demonstrated colocalization of MT1-MMP and MMP9 in osteoclast podosomes. Matrix degradation was observed in areas where podosomes are present [38]. Formation of structurally and functionally linked protease/protein complexes on podosomes dissolves the matrix proteins and paves the way for the migration of osteoclasts. We have previously demonstrated colocalization of CD44 and MMP9 at the migratory front of osteoclasts [20]. Our previous and present studies in osteoclasts knockdown of MT1-MMP and null for CD44 (Figures 5 and 6) indicate that spatial distribution of MMP9, CD44, and MT1-MMP on the cell surface and podosomes functions in a controlled and coordinated manner to digest extracellular matrix proteins enabling osteoclast migration [20, 38]. However, neither polarization nor bone resorption are affected under these conditions (Figure 7). These observations agree with published roles for MMP9 and MT1-MMP (a.k.a. MMP14) in osteoclast migration and not bone resorption [45].

MT1-MMP has now become one of the best characterized enzymes in the MMP family. However, the role of CD44 in the membrane localization of MT1-MMP is not well understood. Our studies have shown the presence of three forms (~63, 55, and 45 kDa) of MT1-MMP in WT osteoclasts. These forms are present in the following order 63 > 45 *⋙* 55 kDa in WT osteoclasts. However, CD44^−/−^ osteoclasts displayed the active form of MT1-MMP (55 kDa) as secreted MT1-MMP in the conditioned medium. Our study with osteoclasts from CD44^−/−^ mice provides molecular evidence that CD44 regulates membrane localization of MT1-MMP. The extracellular portion of MT1-MMP has a catalytic domain (CAT) linked to the hemopexin- (HPX-) like domain through the hinge region [13]. MT1-MMP binds to the extracellular portion of CD44 through the hemopexin- (HPX-)like domain and colocalizes in the cells. Disruption of MT1-MMP/CD44 complex by overexpressing the HPX domain resulted in inhibition of the cleavage and shedding of CD44 [46]. It appears that CD44 plays a key role in assembling MT1-MMP to the cell surface. There also is evidence that activation of MMP9 by an MT1-MMP associated protein occurs through activation of RhoA and actin remodeling [30]. We have previously demonstrated that surface expression of CD44 is linked with actin polymerization and CD44 phosphorylation by RhoA activation [20]. As suggested by others CD44 may be one of the key proteins that interact with MT1-MMP and promote cell migration [16, 30].

CD44 was shown to be shed by proteolytic cleavage by MMPs [47–49]. The shedding was shown to be inhibited by the inhibitors of MMPs or serine proteinases [49]. Earlier observations by Okamoto et al. showed a relationship between proteolytic processing of CD44 by MMPs and transcriptional activation in the nucleus [50]. We have shown here that SiRNA to MT1-MMP reduces total and surface levels of CD44. This indicates that CD44 is a direct target of MT1-MMP in osteoclasts. MT1-MMP and MMP9 are shown to interact with CD44 in different cell systems either directly or indirectly [43, 46, 51, 52]. We have previously demonstrated localization of MMP9 and actin in the sealing ring of resorbing osteoclasts. However, colocalization is negligible [20]. An increase in the secretion of MT1-MMP and a decrease in the active form of MMP9 suggest a possible role for CD44 in the localization of MT1-MMP and MMP9 on the cells surfaces.

Among the MT-MMPs, MT1-MMP is shown to be frequently expressed in highly migratory cells including macrophages, endothelial cells, and invasive cells [7, 16, 53]. We show here that MT1-MMP forms a complex with TIMP2 in CD44^−/−^ osteoclasts. The secreted level and activity of MT1-MMP are more in CD44^−/−^ than WT osteoclasts. However, a significant decrease in the level of TIMP2 was observed in the conditioned medium of CD44^−/−^ osteoclasts as compared with WT osteoclasts. Based on the ratio of MT1-MMP and TIMP2 in the conditioned media, we suggest that the activity of MT1-MMP is tightly regulated by the levels of TIMP2. Low levels of TIMP2 in the conditioned media of CD44^−/−^ osteoclasts correlated with an enhanced activation of MT1-MMP when compared with WT osteoclasts. TIMP2 binds free MT1-MMP at the catalytic site [23]. It has been shown by others that the concentration of 55 kDa MT1-MMP on the cell surface is directly and positively regulated by TIMP2. In the absence of TIMP2, MT1-MMP undergoes autocatalysis to a 44 kDa form [44]. 

The levels of 55 kDa MT1-MMP and TIMP2 are considerably lower in the membrane fraction of WT osteoclasts. Autocatalytic product is seen as 45 kDa in the membrane fraction of WT osteoclasts. Latent MT1-MMP binds TIMP2. Immunoblotting analysis of total membrane fraction consistently shows an MT1-MMP protein with MW ~60 kDa (Figure 1(a), lane 1). It is possible that 60 kDa MT1-MMP contributes to the activity shown in Figure 1(g) in WT osteoclasts. This may function as a latent form of MT1-MMP in WT osteoclasts and possibly did not have affinity to TIMP2. As suggested by others [44] it is possible that differences in the molecular weight of latent MT1-MMP in WT and CD44^−/−^ osteoclasts may be due to differential solubility of these forms when NP-40 containing lysis buffer was used. After surface activation, it may be released into the medium. Since the ratio of MT1-MMP to TIMP2 is less, we presume that MT1-MMP is more active in the conditioned medium than in membrane. Reduced activity in the membrane may be due to interaction of TIMP2 with latent MT1-MMP on the cell surface as shown in Figure 2(b) (left panel). It is possible that differences in the activity of MT1-MMP in WT and CD44^−/−^ osteoclasts may be due to a variation in the ratio of MT1-MMP to TIMP2 (Figure 2). 

 By cross linking experiments, it has been hypothesized that MT1-MMP and TIMP2 form a “receptor” complex that binds MMP2 via its C terminus. High concentrations of TIMP2 inhibit MMP2 activation, because all available MT1-MMP molecules form MT1-MMP/TIMP2 complex. This leads to a condition of no free MT1-MMP molecules to activate MMP2 [41]. MMP2 level is considerably low or not detected at times in the conditioned media of WT or CD44^−/−^ osteoclasts (Figure 1; [20]). MT1-MMP/TIMP2/MMP2 complex formation was not observed in WT osteoclasts (data not shown). Therefore, it is unlikely that MT1-MMP/TIMP2/MMP2 complex is involved in the activation of pro-MMP9 in osteoclasts. MMP3, MMP7, and MMP13 have been shown to activate pro-MMP9 [54, 55]. Although MT1-MMP knockdown regulates pro-MMP9 activation in WT osteoclasts, it is not known whether it directly or indirectly activates pro-MMP9. MT1-MMP knockdown studies suggest MT1-MMP as an upstream regulator of pro-MMP9. To gain further insight into the role of MT1-MMP in the activation of pro-MMP9, future studies should focus on whether MT1-MMP activates pro-MMP9 in cooperation with other MMPs (MMP3, -7, or -13) in osteoclasts. 

## 5. Conclusions

Our data provide molecular evidence that CD44 regulates membrane localization of MT1-MMP. Interestingly, observations in CD44^−/−^ osteoclasts suggest that CD44-TIMP2-MT1-MMP axis regulates MMP9 activity. Surface expression of CD44, localization of MT1-MMP on the cell surface, and activation of pro-MMP9 are the necessary sequence of events in osteoclast migration. Delineating the specific mechanisms that regulate MT1-MMP and consequently MMP9 activity in osteoclasts remains an important goal in our understanding of the function of CD44 on the cell surface.

## Supplementary Material

The effect of different SiRNA (MT1-MMP or MMP9) on the cellular levels of CD44s is shown in supplementary Figure 1. Reduced expression of CD44s (C, lanes 3-4) was observed in cells treated with a SiRNA to MT1-MMP (SR417747). CD44 levels (cellular or surface) were unaffected (D, lanes 2-4; top and bottom panels) in osteoclasts treated with SiRNAs from Origene (B, lane 4) or Ambion (lanes 5 and 6). However, these SiRNAs reduced the levels of MMP9 in these osteoclasts.

## Abbreviations

MMP: Matrix metalloproteinase
MT1-MMP: Membrane type 1 matrix metalloproteinase
HPX: Hemopexin
CD44: Cluster of differentiation (also well known as cell surface glycoprotein and adhesion receptor)
CD44s: Standard CD44
CD44^−/−^ mice: CD44-null mice
WT mice: Wild-type mice
TIMP1 or -2: Tissue inhibitor of metalloproteinase 1 or 2
ECM: Extracellular matrix
SiRNA: Small interference RNA
FITC: Fluorescein isothiocyanate (green-fluorescent fluorescein dye was used)
GM6001: Broad spectrum MMP inhibitor.
