# Supplementary material for: Membrane Localization of Membrane Type 1 Matrix Metalloproteinase by CD44 Regulates the Activation of Pro-Matrix Metalloproteinase 9 in Osteoclasts
Source: Biomed Res Int. 2013 Jul 28;2013:302392. doi: 10.1155/2013/302392 (PMC3745902; doi:10.1155/2013/302392)
Supplement: Supplementary file 1 — The effect of different SiRNA (MT1-MMP or MMP9) on the cellular levels of CD44s is shown in supplementary Figure 1. Reduced expression of CD44s (C, lanes 3-4) was observed in cells treated with a SiRNA to MT1-MMP (SR417747). CD44 levels (cellular or surface) were unaffected (D, lanes 2-4; top and bottom panels) in osteoclasts treated with SiRNAs from Origene (B, lane 4) or Ambion (lanes 5 and 6). However, these SiRNAs reduced the levels of MMP9 in these osteoclasts. [file 302392.f1.docx]

**Supplementary Figure 1.**


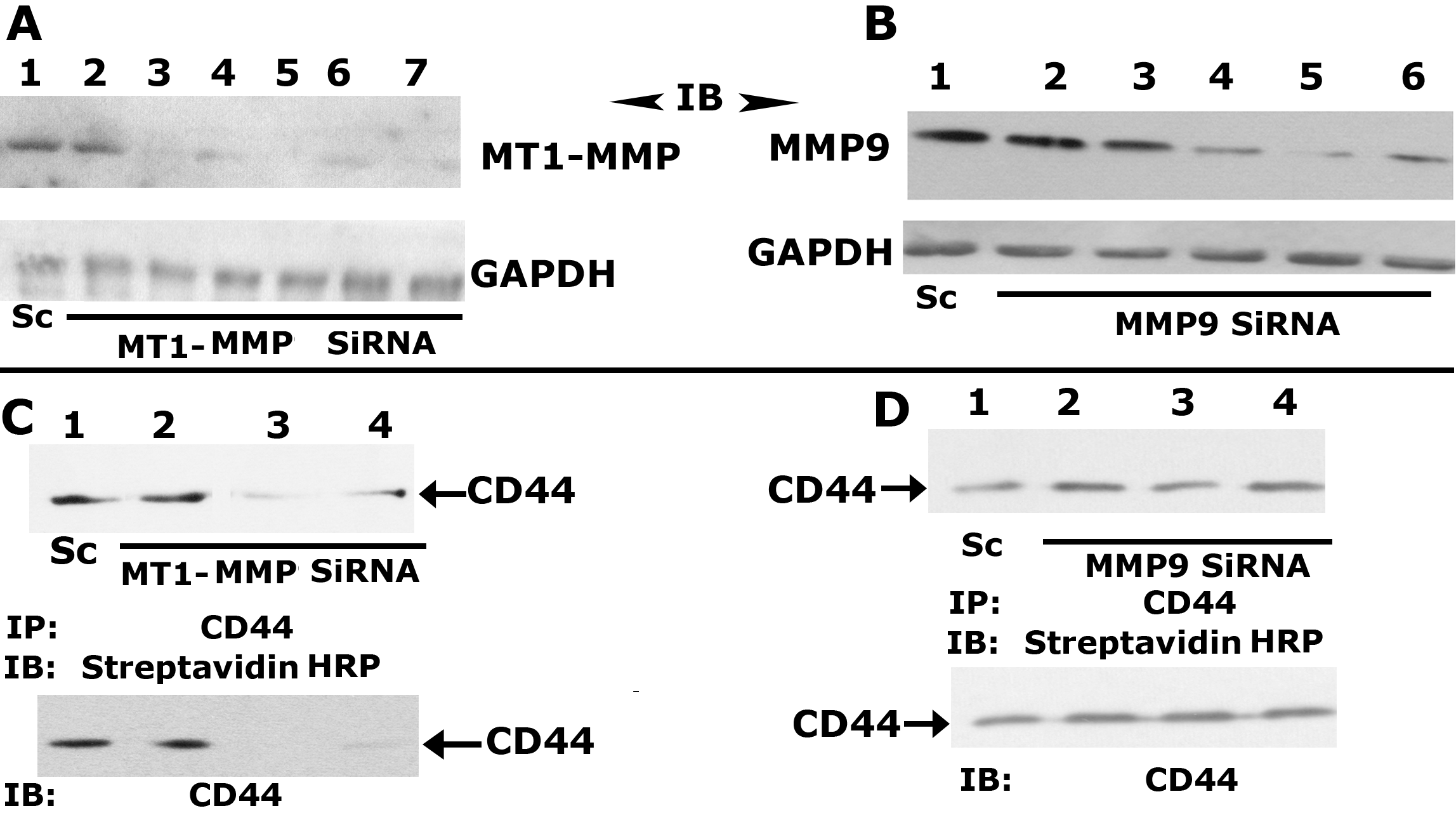


**Method.** SiRNA to MT1-MMP (panel A): Three unique 27mer SiRNA duplexes to MT1-MMP (akaMMP14) was bought from Origene -SR302934 (h); A, lanes 2-4 and SR417747 (m); A; lanes 5-7. SiRNA to MMP9 (panel B): SiRNA duplexes were bought from Origene (SR419619 panel B; lanes 2-4) and also made from Ambion (lanes 5 and 6). Five different duplexes were used (lanes 2-6 in B). Transfection was carried out using the MIRUS transfection reagent according to the manufacturer’s instructions as described previously [1]. After transfection for 18-24h, cells were labeled with NHS-biotin according to the manufacturer's guidelines (Pierce, Rockford, IL). Briefly, cells were incubated with 0.5 mg/ ml biotin for 30–40 min. at 4°C and washed three times with cold PBS. Cells were lysed with RIPA lysis buffer. Equal amounts of protein lysates were used for immunoprecipitation with an antibody to MT1-MMP. The immune complexes were adsorbed onto streptavidin agarose which precipitates the biotinylated MT-MMP protein from the cell surface as described previously [2].

**Result**. Reduced expression of CD44s (C, lanes 3-4) was observed in cells treated with SiRNA to MT1-MMP (SR417747). CD44 levels (cellular or surface) were unaffected (D, lanes 2-4; top and bottom panels) in OCs treated with SiRNAs from Origene (B, lane 4) or Ambion (lanes 5 and 6). However, these SiRNAs reduced levels of MMP9 in these OCs.

1. Chellaiah MA: Regulation of podosomes by integrin alphavbeta3 and Rho GTPase-facilitated phosphoinositide signaling. *Eur J Cell Biol* 2006, 85: 311-317.

2. Chellaiah MA, Kizer N, Biswas R, Alvarez U, Strauss-Schoenberger J, Rifas L *et al*.: Osteopontin Deficiency Produces Osteoclast Dysfunction Due to Reduced CD44 Surface Expression. *Mol Biol Cell* 2003, 14: 173-189.
